# Minimal Cues of Possession Transfer Compel Infants to Ascribe the Goal of Giving

**DOI:** 10.1162/opmi_a_00024

**Published:** 2019-05-01

**Authors:** Denis Tatone, Mikołaj Hernik, Gergely Csibra

**Affiliations:** Cognitive Development Center, Department of Cognitive Science, Central European University; Cognitive Development Center, Department of Cognitive Science, Central European University; 3 Cognitive Development Center, Department of Cognitive Science, Central European University; 4 Department of Psychological Sciences, Birkbeck, University of London

**Keywords:** action understanding, giving action schema, infants, goal ascription

## Abstract

Human infants’ readiness to interpret impoverished object-transfer events as acts of giving suggests the existence of a dedicated action schema for identifying interactions based on active object transfer. Here we investigated the sensitivity of this giving schema by testing whether 15-month-olds would interpret the displacement of an object as an agent’s goal even if it could be dismissed as a side effect of a different goal. Across two looking-time experiments, we showed that, when the displacement only resulted in a change of object location, infants expected the agent to pursue the other goal. However, when the same change of location resulted in a transfer of object possession, infants reliably adopted this outcome as the agent’s goal. The interpretive shift that the mere presence of a potential recipient caused is testament to the infants’ susceptibility to cues of benefit delivery: an action efficiently causing a transfer of object possession appeared sufficient to induce the interpretation of goal-directed giving even if the transfer was carried out without any interaction between Giver and Givee and was embedded in an event affording an alternative goal interpretation.

## INTRODUCTION

Tatone, Geraci, and Csibra (2015) proposed that infants’ ability to represent giving actions is served by a specialized action schema. The function of such a schema is to provide a structural template for the efficient representation of social interactions involving active resource transfer (giving). The schema embeds a set of assumptions about the number and kind of constituents that a giving event should exhibit as well as their causal relations. These assumptions correspond to diagnostic cues that the schema is sensitive to (1) the presence of two agents (a Giver and a Givee) and one object (the transferred item) and (2) a teleological and causal relation between the Giver’s action and the transfer of object possession to the Givee.^1^ When detected, these cues engage the schema, yielding a coherent representation of the event.

Several studies provide evidence of these cues being necessary for infants’ representation of giving. Infants did not expect equal allocations in third-party distributions when shown an agent dividing resources between two inanimate recipients (Geraci & Surian, 2011; Meristo, Strid, & Surian, 2016). Similarly, infants did not show a preference for prosocial characters (Givers) over antisocial ones (Takers), when these interacted with an inanimate patient rather than with an animate patient (Hamlin & Wynn, 2011). Relatedly, infants presented with two agents performing kinematically identical displacing actions ascribed two different goals to these agents depending on whether the displacement resulted in the mere relocation of the object or in a transfer of possession (Tatone et al., 2015). Furthermore, infants habituated to a puppet approaching another while carrying an object detected the removal of the object from the event only if the approach culminated in a transfer of the object, rather than in the two puppets hugging each other (Gordon, 2004; Wellwood, Xiaoxue He, Lidz, & Williams, 2015). These findings illustrate how the assumptions embedded in the giving schema are selective to its representational target (instances of giving), excluding superficially similar, yet functionally different, actions such as disposing of an object or establishing physical contact with another agent. Besides determining the minimal number of event constituents and their respective roles (two agents and one object), the schema also specifies the teleological and causal relations occurring between them. Infants shown an inefficient transfer event, where one puppet carried an object to another puppet but dropped it halfway, failed to encode the agents’ roles as Giver and Givee (Schöppner, Sodian, & Pauen, 2006). Relatedly, infants did not react to two agents having an unequal number of objects if these were not brought about by a distributor, but revealed to be preexisting endowments (Sloane, Baillargeon, & Premack, 2012).

While these findings show that certain cues are necessary for the schema to activate, they also suggest these to be wholly sufficient to this end. Indeed, several of the studies documenting infants’ ability to represent giving actions used impoverished transfer events featuring puppets or simple geometrical agents. Notably, infants readily represented giving-based interactions even when lacking cues of shared attention, communication, or acceptance of the transferred object (Tatone et al., 2015). Infants’ propensity to interpret these skeletal events as giving suggests that cues of possession transfer may suffice by themselves to induce the representation of a giving goal.

Two predictions follow from this hypothesis. First, infants should be prone to interpret an agent efficiently causing a transfer of object possession as having this goal even when this interpretation could be conveniently discounted—for example, because the transfer can be represented as a side effect of the agent’s pursuit of another goal. Second, infants should consider an action as an instance of giving even if the candidate Giver and Givee can be interpreted as causally and teleologically related exclusively on the basis of the change of object possession, but do not otherwise interact with each other. In other words, we hypothesize that, if provided with sufficient cues, infants should be drawn to interpret an event as goal-directed giving despite the availability of competing goal interpretations and the lack of any interaction cue between Giver and Givee besides the transfer itself. Neither of these predictions has been directly tested before: the studies reviewed above always presented infants with actions producing a single outcome of possession transfer following a proximal interaction between Giver and Givee (i.e., one agent approaching another while transporting an object).

Our study aimed at testing both of these predictions. To this end, we contrasted giving with another action known to be easily understood by infants: approaching (Gergely & Csibra, 2003). We also spatially divorced the Giver from the Givee to remove any cue of social proximity from the giving action. Fifteen-month-old infants were presented with an animated event in which an agent simultaneously produced two outcomes: (1) the displacement of an object (A) to a new location (next to another entity) and (2) the approach of a second object (B). Both outcomes resulted from the agent pushing object A, which partly obstructed her path to B. Consequently, infants could interpret the action as directed either at making object A reach a new location, or at freeing the path to B (or could remain undecided between these two options). Depending on the interpretation adopted, the change of A’s location thus constituted either the agent’s goal or a side effect produced while pursuing a different goal.

In order to induce the interpretation of giving, we only manipulated the type of entity next to which the displaced object landed: an inanimate object (Disposing condition) or an animate but motionless agent (Giving condition). If perceiving an efficient possession transfer from one agent to another is sufficient to engage the schema, the introduction of a potential recipient for the displaced object in the Giving condition should induce infants to entertain the distal effects of the pushing action (the relocation of the object next to the second agent) as the agent’s goal—crucially, despite the fact that the same action could be interpreted as aimed at another goal (approaching B).

## EXPERIMENT 1

**Figure 1.**
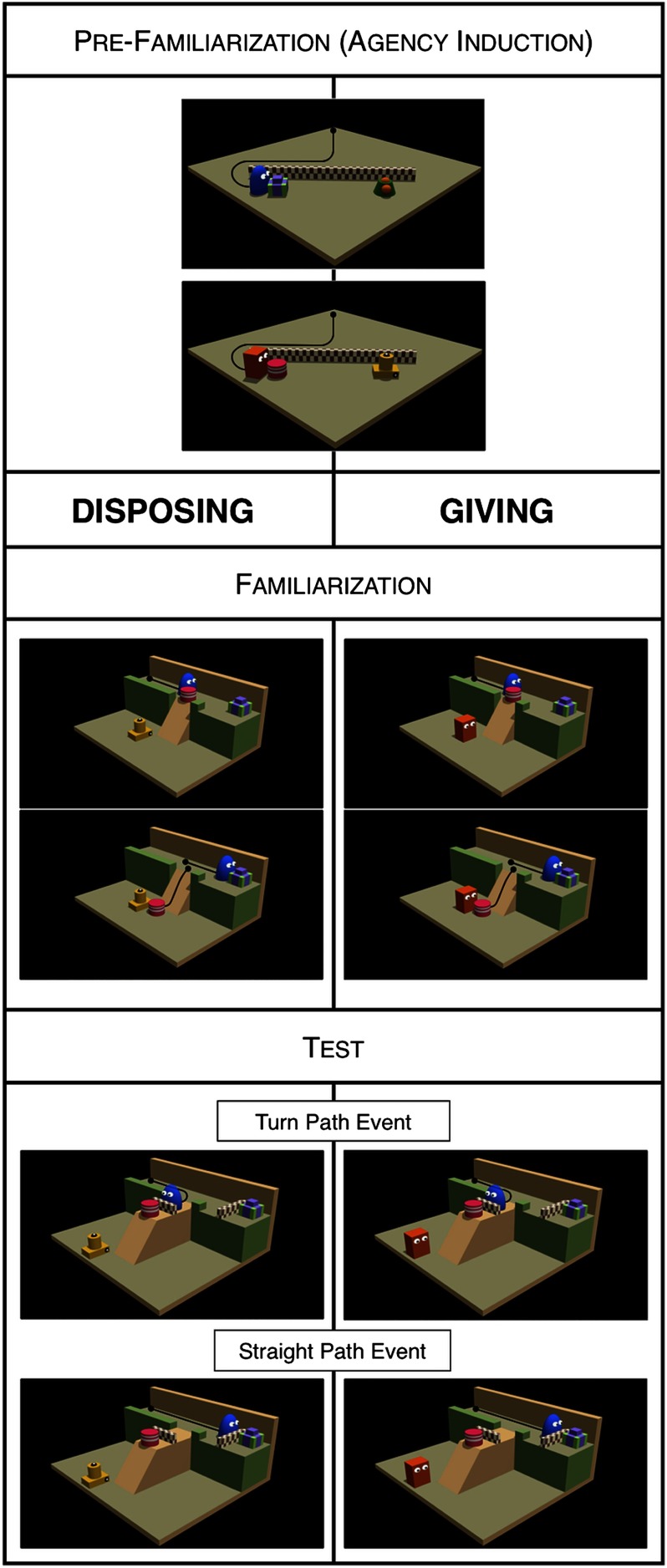
**Schematic visualization of the events shown in Experiment 1.** Black lines indicate the motion paths of agents and objects and were not visible to the infants. The agents’ roles and the identity of the objects were counterbalanced across infants.

The experiment included three phases: pre-familiarization, familiarization, and test. The pre-familiarization served the purpose of inducing the representation of two agents (Blue and Red) as goal-directed. The familiarization event showed Blue moving through a narrow corridor, bumping into object A until pushing it away, and finally arriving at object B.^2^ Infants thus saw an agent producing two distinct outcomes: the relocation of object A and the approaching of object B. Between conditions we only varied the type of entity next to which object A landed: an inanimate object (Disposing condition) or agent Red (Giving condition). Thus, despite the fact that in both conditions the displacement of object A produced a salient change of object location, only in the Giving condition could this outcome have also been interpreted as a transfer of possession. Our main question of interest was whether infants would entertain such an outcome as the goal of the observed action. To answer this question, in the test phase we used a modified landscape, where object A was placed on a lateral platform (so that it could no longer be approached on the way to object B), and presented infants with agent Blue moving toward either object A or object B in two separate trials (Figure 1).

We predicted that, when object A ended up next to another object (Disposing condition), infants should not entertain this outcome as a possible goal state, but should interpret the action as directed at approaching object B. Consequently, at test they should expect agent Blue to take the path directly leading to object B, since acting on object A would be no longer required to achieve this outcome. In contrast, when the displacement of object A resulted in a possession transfer (Giving condition), infants should entertain this outcome as an additional goal state. Because of this, we predicted that infants would have no specific expectations about which of the two objects agent Blue would approach, since both would be compatible with either of the goal hypotheses formed (giving object A or approaching object B). Thus, we expected infants in the Giving condition to no longer privilege the approach of object B as Blue’s goal.

### Methods

#### Participants.

Thirty-two 15-month-olds participated in the experiment, half in the Giving condition, the other half in the Disposing condition. The mean age of the final sample was 464 days (range: 456–472 days) in the Giving condition and 476 days (range: 452–479 days) in the Disposing condition. An additional 12 infants were excluded from the analysis for crying during the test phase (*n* = 2), inattentiveness (*n* = 5), and experimenter error (*n* = 5). The sample size was pre-fixed at 16 participants per condition by estimating the effect size of previous studies using looking-time measures with similar age groups and procedures (e.g., Mascaro & Csibra, 2014; for details of estimation, see Csibra, Hernik, Mascaro, Tatone, & Lengyel, 2016).

#### Stimuli.

The stimuli were computer-generated animations designed in Blender (version 2.59) and presented using Keynote on a LCD screen (40-inch diagonal). Besides landscape items, two self-propelled agents (Blue and Red) and four inert objects (A, B, C, and D) were featured in these animations. For a detailed description of stimuli appearance, timing, and counterbalancing see the Supplemental Materials (Tatone, Hernik, & Csibra, 2019).

##### *Pre-familiarization*.

The events started with an agent (Blue or Red) and two target objects (B and C, or A and D, respectively) placed behind a short wall. In each trial, the agent approached selectively one of the two objects. There were eight trials in two four-trial blocks. In one block, agent Blue approached object B; in the other, agent Red approached object A. Within these blocks, the two target objects swapped locations for the third and fourth trials. Each event lasted 6.35 s, with the last frame kept for an additional 4.5 s, and was automatically terminated upon reaching its end.

##### *Familiarization*.

The event started with agent Blue at one end of a narrow corridor, oriented toward object B placed at the opposite end. At the corridor’s midpoint, a slope directly connected the corridor to a lower platform. Object A was placed on the slope’s edge, partly obstructing agent Blue’s access to object B. Depending on the condition, there was either agent Red (Giving) or object D (Disposing) at the bottom of the slope. The event showed agent Blue moving through the corridor until bumping into object A, displacing it, and proceeding toward object B. Once slid off the slope, object A fell in the proximity of the entity placed below the slope. Each event lasted 5 s with the last frame kept still for an additional 5 s, and was automatically terminated upon reaching its end.

##### *Test events*.

These events involved the same characters and a similar landscape as those used during familiarization. The slope, rather than being directly attached to the corridor, was connected to a lateral platform branching out from it. As a result of this, agent Blue could now direct its approach only toward one of the two objects (A or B) at a time. Neither of these objects could be fully reached because of the presence of a short wall in front of each. There were two types of test events: in the Straight Path event, agent Blue moved straight until contacting the wall in front of object B, whereas in the Turn Path event, agent Blue turned to the lateral corridor until contacting the wall in front of object A.

#### Procedure.

The infants were tested in a dimly lit and soundproof room. They sat on the parent’s lap, 100 cm away from the presentation screen. A hidden camera mounted under the screen recorded infants’ looking behavior at 25 frames per second. The parents were instructed to keep their eyes closed during the whole procedure. The infants were first presented with eight pre-familiarization trials (the same in both conditions), followed by four familiarization trials (differing between conditions only in the “recipient” of the displaced object), and 2 test trials (Straight Path and Turn Path) in counterbalanced order.

#### Coding and data analysis.

We coded looking behavior frame-by-frame off-line. Looking time during test trials was measured from when the agent reached one of the two walls to the moment when the infant looked away for more than 2 s or looked cumulatively for 60 s (for more information, see the Supplemental Materials [Tatone et al., 2019]). Parametric statistics were performed on log-transformed looking time data (Csibra et al., 2016). We show untransformed looking times in the figures.

### Results

**Figure 2.**
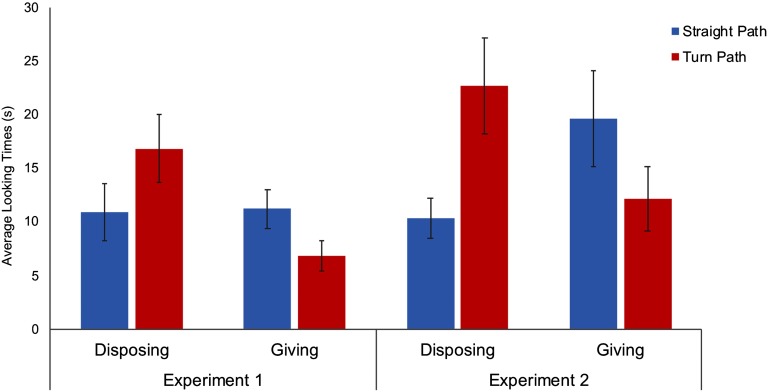
**Average looking times during the test trials as a function of conditions in Experiment 1 and 2.** Error bars indicate standard errors.

There was no difference between conditions in how long infants attended to the familiarization events, *F*(1, 31) = 0.789, *p* = .379. An ANOVA on the looking times in the test trials with trial type (Straight vs. Turn Path) as a within-subject factor and Condition (Disposing vs. Giving) as a between-subject factor revealed no main effect, but an interaction, *F*(1, 30) = 9.25, *p* = .005, *η*_*p*_^2^ = .236. The infants in the Disposing condition looked longer at the Turn Path than at the Straight Path test trial, *p* = .014 by Wilcoxon signed-rank test; *F*(1, 30) = 4.10, *p* = .052 by planned contrast. On the contrary, the infants in the Giving condition looked longer at the Straight Path than at the Turn Path test trial, *p* = .026 by Wilcoxon signed-rank test; *F*(1, 30) = 5.18, *p* = .030 by planned contrast (Figure 2). At an individual level, 13/16 infants in the Disposing condition looked longer at the Turn Path test event, whereas 12/16 infants in the Giving condition showed the opposite looking pattern (*p* = .004 by Fisher’s exact test).

### Discussion

As predicted, the infants in the Disposing condition looked longer at the Turn Path test event, indicating that they interpreted the agent’s actions during familiarization as directed at reaching object B. This finding validated the use of the familiarization event to induce goal attribution and provided a benchmark of comparison for assessing whether introducing an agent as the potential recipient of object A would cause infants to interpret the pushing action as directed to causing a possession transfer. As the looking-time reversal in the Giving condition showed, the presence of agent Red proved sufficient to induce such an interpretive shift. In fact, the presence of this giving cue exerted an even greater influence than originally predicted: rather than entertaining both outcomes as possible goal states, the infants in the Giving condition reliably expected agent Blue to approach object A, apparently disregarding the alternative goal that the infants in the Disposing condition attributed to agent Blue.

Crucially, the same cues that prompted infants to interpret the displacement of object A as functional to the approach of object B in the Disposing condition were similarly available in the Giving condition. In both, agent Blue showed persistence in its goal-directed approach toward object B, by bumping repeatedly against the obstructing object; it kept its orientation fixed toward this object throughout the motion; and, most importantly, it reached for an object featurally identical to the one selectively approached during pre-familiarization. It is thus remarkable that simply replacing the inanimate recipient with an animate, yet motionless, one made infants favor the interpretation of the agent’s action as giving over a goal hypothesis that, absent this single cue of giving, the familiarization events reliably supported.

## EXPERIMENT 2

The aim of Experiment 2 was twofold. First, given the unexpected reversal of the looking-time pattern in Experiment 1, we decided to assess the robustness of these findings via a replication. Second, we tested whether information about the value of the transferred object may have contributed to the interpretive shift observed between conditions. The fact that the displaced object (A) was featurally identical to the one approached by the recipient (agent Red) during pre-familiarization leaves open the possibility that infants may have perceived the pushing action as causing not only the transfer of an object, but specifically of an object previously inferred to be valuable for the recipient. Under our account, information about object value should not have played a role in favoring the giving interpretation, since the action itself exhibited sufficient cues to engage the schema.

To test whether infants would interpret the possession transfer as the agent’s goal, absent any prior relation between the recipient and the transferred object, in Experiment 2 we modified the pre-familiarization events so that agent Red approached a different object from the one later displaced.

### Methods

Experiment 2 differed from Experiment 1 only in one respect. In the pre-familiarization phase agent Red always approached object D rather than A (Table 1), thus ensuring that the object transferred to agent Red during familiarization would be different from the one earlier approached.

**Table 1.** The distribution of agents (Blue, Red) and objects (A, B, C, D) as a function of Experiment and Condition. The agents’ roles and the identities of the approached objects were counterbalanced across infants.

	**Experiment 1**		**Experiment 2**	
	**Disposing**	**Giving**	**Disposing**	**Giving**
**Pre-familiarization**	Blue approaches B over C		Blue approaches B over C	
	Red approaches A over D		Red approaches D over A	
**Familiarization**	Blue transfers A to D and approaches B	Blue transfers A to Red and approaches B	Blue transfers A to D and approaches B	Blue transfers A to Red and approaches B
**Test**	Blue approaches A		Blue approaches A	
	Blue approaches B		Blue approaches B	

#### Participants.

Thirty-two 15-month-olds participated in the experiment. The mean age of the infants in the final sample was 469 days (range: 454–477 days) in the Giving condition and 467 days (range: 452–479 days) in the Disposing condition. An additional 11 infants were excluded from the analysis for inattentiveness (*n* = 7), crying (*n* = 1), and experimenter error (*n* = 3).

### Results

There was no difference between conditions in how long 15-month-olds attended to the familiarization events, *F*(1, 31) = 0.773, *p* = .386. An ANOVA ran in the same way as in Experiment 1 on the looking times to the test events revealed only an interaction between trial type and Condition, *F*(1, 30) = 7.79, *p* = .004, *η*_*p*_^2^ = .206. As in Experiment 1, the infants in the Disposing condition looked longer at the Turn Path than at the Straight Path test trial, *p* = .003 by Wilcoxon signed-rank test; *F*(1, 30) = 9.04, *p* = .005 by planned contrast. On the contrary, the infants in the Giving condition looked longer at the Straight Path than at the Turn Path test trial, *p* = .030 by Wilcoxon signed-rank test (Figure 2). However, this effect was not significant by parametric analysis: *F*(1, 30) = 0.94, *p* = .354 by planned contrast. The pattern of results was also confirmed by the fact that 12/16 infants in the Disposing condition looked longer to the Turn Path test event, whereas 11/16 infants in the Giving condition exhibited the opposite looking pattern (*p* = .034 by Fisher’s exact test).

Given the lack of significance of the parametric analysis within the Giving condition of Experiment 2, we sought to assess the consistency of infants’ reactions to the Disposing and Giving test events between experiments. We performed an omnibus ANOVA with Experiment (1 vs. 2) and Condition (Giving vs. Disposing) as between-subject factors and test trial type (Straight vs. Turn Path) as a within-subject factor. The analysis revealed a strong interaction between test trial type and Condition, *F*(1, 60) = 18.64, *p* < .001, *η*_*p*_^2^ = .237, and no main effect of, or interaction with, Experiment.

### Discussion

Experiment 2 closely replicated the results of Experiment 1. The infants disregarded the change of an object location as a potential goal state when it did not result in a possession transfer, whereas they prioritized its ascription when it did. Importantly, the similarity of findings across experiments supports the conclusion that information about the value of the object transferred to the recipient is unnecessary to represent this action as an instance of goal-directed giving.

## GENERAL DISCUSSION

Across two experiments, we found that 15-month-old infants spontaneously interpreted the displacement of an object to a new location as the agent’s goal when the object ended next to another agent, whereas they disregarded this outcome in favor of an alternative goal hypothesis when the object ended next to another object. By merely introducing a potential recipient for the displaced object we were able to flip infants’ interpretation of an outcome from a teleologically irrelevant side effect to the agent’s goal. The occurrence of such shift in spite of the presence of a competing goal hypothesis provides a striking demonstration of the interpretive pull that minimal cues of giving have on infants’ action interpretation.

In fact, these cues exerted an even greater influence than originally predicted: they did not induce infants to merely entertain a second goal hypothesis (giving object A) alongside the one that the event was designed to invite (approaching object B), but made them consistently privilege the former over the latter, as the looking-time reversal between conditions demonstrates. We consider two possible explanations for this finding. The first one proposes that when infants cannot adjudicate among multiple outcomes which is the agent’s goal on the basis of cost information alone, they assign goal status to the outcome inferred to produce higher benefits for the agent, following the normative principle of utility maximization (Jara-Ettinger, Gweon, Schulz, & Tenenbaum, 2016). Applied to our study, which featured two outcomes brought about in a comparably efficient manner, this account proposes that our participants prioritized the ascription of the giving goal because it generated higher utility than the alternative goal state (approaching object B). This interpretation may seem paradoxical at first, since giving by definition entails net costs for the donor. Such a paradox, we contend, is only apparent if prospective benefits can be expected to accrue to the donor as a consequence of her altruistic act. One source of such benefits, which young children take into account when deciding whom to share with or request resources from (Olson & Spelke, 2008; Paulus, 2016), consists in future reciprocation by past beneficiaries. Under this reading, the higher utility of the giving outcome, which turned it into a privileged goal state, reflects infants’ appreciation of the long-term gains of reciprocal exchanges, established via resource donation.

While this account postulates that two distinct goal hypotheses were concurrently activated in the Giving condition, it may also be possible that the presence of giving-diagnostic cues made infants completely disregard further effects of the agent’s action. This account may also imply that, having interpreted the agent’s action during familiarization as only directed at giving, infants assessed the agent’s behavior at test exclusively in relation to this goal’s satisfaction. Thus, the Straight Path event might have elicited longer looking not because the agent pursued a different goal from the one attributed, but because it selected a different object for the recipient from the one previously transferred.^3^ Whichever the mechanism through which infants conferred goal status to the displacement outcome, both accounts presuppose that the presence of minimal cues of possession transfer was sufficient to produce such attribution.

It is worth pointing out how skeletal was the implementation of giving we opted for in this study. First, other than its proximity to the transferred object, infants had no additional cue to represent the second agent as recipient. The agent was motionless throughout the transfer, exhibiting none of the requesting or acknowledging behaviors that accompany real-life instances of giving (Hay & Murray, 1982). More importantly, because of the spatial separation of the two agents, infants had no other cues to represent the Giver’s action as related to the other agent than the transfer of possession. Yet, the relocation of the object next to a potential beneficiary, caused at distance by an agent showing no manifest behavioral sign of concern for the object’s fate after its displacement, proved sufficient to induce the interpretation of the event as goal-directed giving. Infants’ susceptibility to minimal cues of possession transfer provides a vivid illustration of the giving schema as a type of “cognitive attractor”: once engaged, the schema slots its event constituents into specific roles (Giver, object, Givee) and interrelates them in a coherent goal representation. While we do not know whether such effects would obtain beyond the conditions here tested, our results demonstrate that, in the presence of the necessary minimal cues, infants are biased to interpret an action as directed at giving even when competing goal interpretations are readily available.

## FUNDING INFORMATION

This research was supported by a European Research Council Advanced Investigator grant (#742231 “PARTNERS”) to GC. The work of MH is supported by an ERC Synergy grant (#609819 “SOMICS”).

## AUTHOR CONTRIBUTIONS

DT: Conceptualization: Lead; Investigation: Lead; Methodology: Lead; Writing – Original Draft: Lead; Writing – Review & Editing: Lead. MH: Data curation: Supporting; Investigation: Supporting; Writing – Review & Editing: Supporting. GC: Conceptualization: Supporting; Data curation: Supporting; Investigation: Supporting; Methodology: Supporting; Supervision: Lead; Writing – Review & Editing: Supporting.

## Notes

^1^ We define possession as an agent’s dispositional ability to control an object relatively to other agents and operationalize it in terms of the agent’s relative proximity to an object (for details, see Tatone et al., 2015).^2^ The roles of Blue and Red were counterbalanced across infants. However, for ease of reading, we refer to the active agent as Blue and the passive one as Red in the article, and depict the events accordingly in Figure 1.^3^ We thank the editor for having suggested this alternative interpretation of the findings.
